# Olfactory mucosa mesenchymal stem cells alleviate pulmonary fibrosis via the immunomodulation and reduction of inflammation

**DOI:** 10.1186/s12890-023-02834-5

**Published:** 2024-01-05

**Authors:** Ran Duan, Chun-Gu Hong, Xin Wang, Ming Lu, Hui Xie, Zheng-Zhao Liu

**Affiliations:** 1 Guangdong Provincial Key Laboratory of Autophagy and Major Chronic Non-Communicable Diseases, Key Laboratory of Prevention and Management of Chronic Kidney Disease of Zhanjiang City, Institute of Nephrology, Affiliated Hospital of Guangdong Medical University, 524001 Zhanjiang, Guangdong China; 2 Department of Sports Medicine, Xiangya Hospital, Central South University, 410008 Changsha, Hunan China; 3 Movement System Injury and Repair Research Center, Xiangya Hospital, Central South University, 410008 Changsha, Hunan China; 4 Department of Neurosurgery, Second affiliated Hospital of Hunan Normal University (921 Hospital of PLA), 410081 Changsha, Hunan China

**Keywords:** Olfactory mucosa mesenchymal stem cells, Pulmonary fibrosis, Monocyte, Inflammation, Collagen

## Abstract

**Background:**

Pulmonary fibrosis (PF) is a progressive fibrosing interstitial pneumonia that leads to respiratory failure and other complications, which is ultimately fatal. Mesenchymal stem cells (MSCs) transplant is a promising strategy to solve this problem, while the procurement of MSCs from the patient for autotransplant remains a challenge.

**Methods:**

Here, we presented olfactory mucosa mesenchymal stem cells (OM-MSCs) from mouse turbinate and determined the preventing efficacy of allotransplant for PF. We demonstrated the antiinflammation and immunomodulatory effects of OM-MSCs. Flow cytometric analysis was used to verify the effect of OM-MSCs on monocyte-derived macrophage populations in the lung.

**Results:**

Administration of OM-MSCs reduces inflammation, attenuates the matrix metallopeptidase 13 (MMP13) expression level and restores the bleomycin (BLM)-induced pulmonary fibrosis by assessing the architecture of lung, collagen type I; (COL1A1), actin alpha 2, smooth muscle, aorta (ACTA2/α-SMA) and hydroxyproline. This therapeutic effect of OM-MSCs was related to the increase in the ratio of nonclassical monocytes to proinflammatory monocytes in the lung.

**Conclusions:**

This study suggests that transplant of OM-MSCs represents an effective and safe treatment for PF.

**Supplementary Information:**

The online version contains supplementary material available at 10.1186/s12890-023-02834-5.

## Background

Pulmonary fibrosis (PF) is a chronic interstitial lung disease characterized by a progressive and irreversible decline in lung function [1]. It is a serious threat to human health because of its complex etiology and poor clinical treatment effect [2]. Its clinical features include shortness of breath, hypoxemia, radiographically obvious lung infiltration and continuous accumulation of fibrosis [3]. However, due to delayed diagnosis of PF and the occurrence of complications, its treatment outcomes and prognosis remain grim [4, 5]. PF possesses intricate and not yet completely comprehended pathogenesis, wherein the intricate interplay of genetic and environmental factors influences various cell types, mainly manifested through epithelial-mesenchymal transition (EMT) and proliferation of myofibroblasts [6–9]. Kinase inhibitors targeting inflammatory signaling pathway, such as Nintedanib or Pirfenidone, may slow the progress of PF with limited therapeutic effect [10]. There is currently no cure for PF. A lung transplant may be an option for some people who have advanced PF [11].

Furthermore, the pathogenesis of PF has not been fully elucidated. It is accepted that pulmonary fibrosis is related to abnormal wound healing associated with immune inflammatory injury. Its main pathological changes include severe destruction of the alveolar structure, massive proliferation of fibroblasts, and extensive extracellular matrix (ECM) deposition. Immune cells and a number of chemokines, such as transforming growth factor, beta 1 (TGFB1), platelet derived growth factor, B polypeptide (PDGFB), tumor necrosis factor (TNF), interleukin 1 beta (IL1B), etc., mediate these pathological changes [12]. Studies by Zhang et al. suggested that inflammation and cytokines promoted the development of fibrosis, due to the accumulation of LY6C1^hi^ (lymphocyte antigen 6 complex, locus C1) macrophages. The exacerbation of pulmonary fibrosis may be relieved by reducing the inflammation state [13–15]. Instead of lung transplant, which has complications of infection and rejection of the new organ, cell transplant is optional in clinical to avoid difficulty of organ procurement. Cell-based therapies like mesenchymal stem cells (MSCs) and other stem cells are studied, showing improved conditions, however, the PF patients must take medicines for the rest of life to reduce the risk of rejection. Recently, reports from different groups showed that olfactory mucosa mesenchymal stem cells (OM-MSCs) own the characteristics of stem cells, maintain the stemness through the whole life, and have beneficial effects for curing nervous system diseases [16–19]. These results along with the advantage that OM-MSCs have the ability of in vitro proliferation, prompt us to test whether the transplant of OM-MSCs would relieve the PF.

Here, we studied the transplant of MSCs isolated from mouse olfactory mucosa into a mouse model of bleomycin (BLM)-induced pulmonary fibrosis to determine whether the fibrosis could be relieved by OM-MSCs transplant. To further explore the role of inflammation in OM-MSCs transplant, we compared the inflammation status of mice transplanted with OM-MSCs and non-transplanted with OM-MSCs, and demonstrated the role of macrophages in OM-MSCs treated PF. The aim of our study was to evaluate the therapeutic efficacy of OM-MSCs in a model of bleomycin-induced pulmonary fibrosis. Compared to mesenchymal stem cells derived from adipose and bone marrow sources, OM-MSCs are easier to obtain and are ideally positioned for autologous transplantation.

## Methods

### Ethics statement and animal handling

This study was approved by the ethical review board at Xiangya hospital of Central South University (No. 2,017,121,175). All procedures for animal use were followed with the guidelines of animal welfare set by the World Organization for Animal Health and the Chinese national guideline for animal experiments. The seven-week-old C57BL/6 male mice, weighing about 20 g, were purchased from SJA laboratory animal center (Changsha, Hunan). The mice were maintained in an air-conditioned animal facility under constant temperature and humidity conditions.

### Mouse OM-MSC preparation for allotransplantation

Olfactory mucosa was isolated from C57BL/6 male mice (purchased from SJA Laboratory Animal Center, Changsha, Hunan) for 3–4 weeks used for OM-MSC collection as described previously [20]. After anesthesia with 0.3% pentobarbital sodium (20 µL/g), mice were decapitated and then we removed the skin, the lower jaw and the bone covering the nasal cavity. Forceps were used to separate the olfactory mucosa from the septum. We transferred the olfactory mucosa to DMEM/F12-filled petri dishes and removed the olfactory epithelium under a microscope. The remaining lamina propria after removal of the mucosal epithelium was then shredded and transferred to medium filled with 0.5% collagenase II. Following incubation of the pellet for 1 h, the cells were resuspended by centrifugation and inoculated into petri dishes containing 10% fetal bovine serum and DMEM/F12 culture medium. Every three days, the medium was replaced. Within a week, stem cells were visible crawling out. Isolated OM-MSCs were stained with PE-conjugated anti-CD73 (BD, 550,741), APC-conjugated anti-CD90 (R&D, FAB7335R) and anti-CD105 (BD, 562,761), FITC-conjugated anti-CD31 (R&D, FAB3628G), anti-CD34 (BD, 560,238), and anti-CD45 (BD,553,079) after in vitro culture expansion. We analyzed 10^5^ events (BD, FACS Canto II) per sample using FlowJo V10.5.3 software and compared them with an isotype control in order to determine the cell gating. For transplantation, cells were subcultured up to passage 3.

### BLM-induced pulmonary fibrosis model

Mice were anesthetized and received a single endotracheal dose of bleomycin (50 µL, 1 mg/kg of mouse body weight; Nippon Kayaku Co.) diluted in phosphate-buffered saline (PBS) to induce pulmonary fibrosis. Seven-week-old male mice were randomly divided into three groups (n = 16 per group): (1) Control group: mice received an endotracheal dose of PBS (50 µL) instead of BLM treatment; (2) BLM + PBS group: BLM-induced mice without OM-MSCs treatment; (3) BLM + OM-MSCs group: BLM-induced mice with OM-MSCs treatment. OM-MSCs (5 × 10^5^ equivalents) were administered by intravenous injection to the tail vein on the 5, 10, 15 days after BLM administration, and PBS was injected as a negative control. On day 21, mice were sacrificed and their lungs were scanned by µCT to evaluate the severity of pulmonary fibrosis. The weights of mice were recorded every other day after BLM treatment.

### Fluorescence imaging

To examine the distribution of OM-MSCs after intravenous, the OM-MSCs were labeled with the DiR Iodide dye (Yeasen, Shanghai, China) for ex vivo fluorescence imaging. In brief, cells were incubated with dye and injected into mice through the tail vein (5 × 10^5^ equivalents). Control mice were treated with an equal volume of solvent. Mice were sacrificed after 6 and 24 h for tissue collection, and fluorescent signals in these tissues were immediately detected by a fluorescence tomography imaging system (FMT-4000; PerkinElmer, USA).

### Histochemistry and immunohistochemical staining

After OM-MSCs treatment for 21 days, left lungs were harvested, fixed with 4% paraformaldehyde, dehydrated by graded ethanol, embedded in paraffin, and sliced into 4-μm-thick sections. Lung structures were assessed by H&E staining. Collagen deposition was determined by Masson’s trichrome stain. Right lungs were frozen at -80° for the isolation of RNA and proteins, and the determination of hydroxyproline content. The modified Ashcroft score was used for quantitative histological analysis by visual assessment of fibrotic changes induced by BLM [21, 22]. Five fields within each lung section were observed, and the score of fibrosis ranged from 0 (normal lung) to 8 (total fibrous obliteration of the field). Scores of 0-1 represented no fibrosis, scores of 2-3 represented minimal fibrosis, scores of 4–5 were considered as moderate fibrosis, and scores of 6-8 indicated severe fibrosis. For Immunohistochemical staining, lung tissue sections were dewaxed in xylene and rehydrated with ethanol of different concentration gradients. Tissue sections were incubated in 3% H_2_O_2_ at room temperature to eliminate endogenous peroxidase activity. Then the slides were microwave-heated in 10 mM citrate buffer and blocked with 10% goat serum for 60 min. Samples were then incubated at 4 °C overnight with the recommended dilution of the primary antibodies including anti-collagen I antibody (1:500, Abcam; ab21286), anti-ACTA2 antibody (1:200, Abcam; ab32575), anti-MMP13 antibody (1:200 Bioworld; BS1231P), anti-TGFB1 antibody (1:200, ZEN-BIOSCIENCE; 346,599) and anti-IL1B antibody (1:500, Abcam; ab9722). Samples were further incubated with horseradish peroxidase- conjugated secondary antibodies (1:300, Servicebio, gb23303) for 1 h. Observe the samples after color development with 3,3’-diaminobenzidine (DAB).

### Hydroxyproline content determination

The content of collagen in the lung is reflected by measuring the content of hydroxyproline in the lung. Quantify the content of hydroxyproline in lung tissue using a commercial hydroxyproline assay kit (Nanjing Jiancheng Bioengineering Institute, Nanjing, China) according to the manufacturer’s protocol.

### µCT analysis

Mice were killed and analyzed by high-resolution µCT (VIVACT 80; SCANCO Medical AG, Switzerland). The scanner was set to 45 kV and 177 µA at 11.4 μm resolution. The data analysis software (CTAn v1.9 and Data Viewer) was applied to analyze the acquired images. Bony chest cage (sternum in the front, vertebrae in the back and ribs on the sides) and trachea lumen (as a small dark circle at the level of the neck and upper chest, which bifurcates into the right and left main bronchi then continues to branch into smaller and smaller bronchi.) were identified. The heart was in front of the chest and the major blood vessels near the heart and in the mediastinum.

### RNA extraction and qRT-PCR analyses

Total RNA was extracted from mouse lung tissues using the standard Trizol method (Takara, Beijing, China). For gene expression analysis, synthesis of cDNA was performed using GoScript™ Reverse Transcriptase according to the manufacturer’s instruction (Promega, A5001). Primers were synthesized in the Beijing Genomics Institute (Beijing, China). QRT-PCR amplification of indicated genes was performed using GoTaq® qPCR Master Mix (Promega, A6001) on an FTC-3000 real-time PCR machine (funglyn biotech) with *Gapdh* as a normalization control. After the initial denaturation (2 min at 95°C), amplification was performed with 40 cycles of 15 s at 95°C and 60 s at 60°C. The sequences of the primers used for qPCR are listed below: m-*Gapdh*-F: 5’-AGGTCGGTGTGAACGGATTTG-3’,

m-*Gapdh*-R: 5’-TGTAGACCATGTAGTTGAGGTCA-3’,

m-*Col1a1*-F: 5’-GCTCCTCTTAGGGGCCACT-3’,

m-*Col1a1*-R: 5’-CCACGTCTCACCATTGGGG-3’,

m-*Acta2*-F: 5’-GTCCCAGACATCAGGGAGTAA-3’,

m-*Acta2*-R: 5’-TCGGATACTTCAGCGTCAGGA-3’,

m-*Mmp13*-F: 5’-CTTCTTCTTGTTGAGCTGGACTC-3’,

m-*Mmp13*-R: 5’-CTGTGGAGGTCACTGTAGACT-3’,

m-*Tgfb1*-F: 5’-TTGCTTCAGCTCCACAGAGA-3’,

m-*Tgfb1*-R: 5’-TGGTTGTAGAGGGCAAGGAC-3’,

m-*Il1b*-F: 5’-AAGGAGAACCAAGCAACGACAAAA-3’,

m-*Il1b*-R: 5’-TGGGGAACTCTGCAGACTCAAACT-3’.

### Western blot

Total proteins from lung tissue were extracted with RIPA lysate containing protease inhibitors (Cwbio, Jiangsu, China) and their concentration was determined using a BCA protein colorimetric assay kit (Elabscience, Wuhan, China). A total of 25 µg protein from each sample was separated by sodium dodecyl sulfate-polyacrylamide gel electrophoresis (SDS-PAGE) and transferred onto polyvinylidene difluoride (PVDF) membranes. After blocking with 5% fat-free milk in TBST for 1 h at room temperature, the membranes were incubated overnight at 4 °C with the following primary antibodies: anti-collagen I (1:250, Abcam; ab21286), anti-ACTA2 (1:1000, ZEN-BIOSCIENCE; 380,909), anti-MMP13 (1:1000, ZEN-BIOSCIENCE; 820,098), anti-TGFB1 (1:1000, ZEN-BIOSCIENCE; 346,599) and anti-IL1B (1:5000, Abcam; ab9722). β-Actin was used as an internal control. The membranes were washed with TBST for three times and then incubated with secondary antibodies (1:5000, Servicebio, gb23303), for 1 h at room temperature, and then exposed to radiography film.

### Flow cytometry

For flow cytometry, lungs were harvested on days 21. Lungs were cut into small pieces and digested in 5 mL of digestion buffer consisting of RPMI-1640 (Biological Industries), collagenase IV (1.6 mg/mL, Worthington Biochemical Corp), and DNase1 (50 unit/mL, Worthington Biochemical Corp). Lungs were shaken at 37 °C for 30 min, and RBCs were lysed using RBC lysis buffer (Solarbio). Homogenized lung was passed through a 70 μm cell strainer (Biologix) to obtain a single-cell suspension. Cells were washed twice with cold PBS and centrifuged at 300 g for 8 min at 4 ℃. 1 × 10^6^ cells were resuspended in 100 µL of cold PBS per sample and stained with APC-conjugated anti-LY6C1 (Thermo, 17-5932-82) and anti-CCR2 (Abcam, ab216863). Primary antibody CCR2 further incubated with Cy3-conjugated goat anti-rabbit secondary antibody (Abcam, ab97075). One hundred thousand events per sample were collected (BD, FACS Canto II), and data were analyzed with FlowJo V10.5.3 software. Cell gating was based on the comparison with isotype control.

### Statistical analysis

All statistical data were presented as mean ± SD. Statistical analyses were using GraphPad Prism 8 software. One-way ANOVA followed by Bonferroni *post hoc* test was used to analyze multiple-group comparisons. * *P* < 0.05, ** *P* < 0.01, *** *P* < 0.001, **** *P* < 0.0001.

## Results

### Administration of OM-MSCs protects lungs from BLM-induced pulmonary fibrosis

**Fig. 1:**
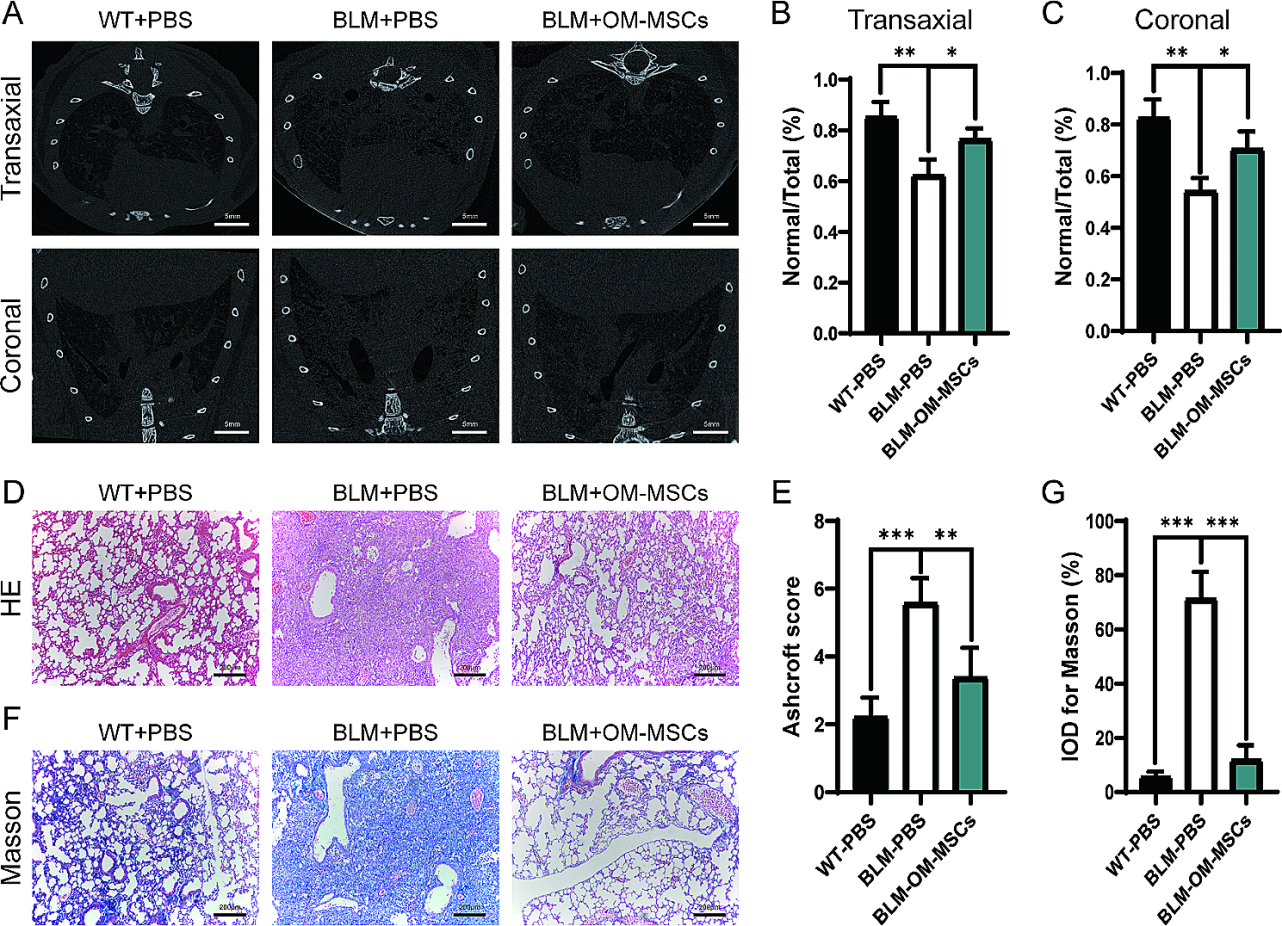
Administration of OM-MSCs protects lungs from BLM-induced pulmonary fibrosis. (**A**). Representative µCT images show the lung architecture of control mice or BLM-induced mice with or without OM-MSCs treatment. Scale bars: 5 mm. n = 3 per group. (**B-C**) Ratio of normal lung tissue to total lung tissue area of transaxial and coronal. (**D-E**) Representative images of HE staining and Ashcroft score. Scale bars: 200 μm. n = 5 per group. (**F-G**) Masson’s trichrome staining images of lung and quantification of the IOD for Masson-stained areas. Scale bars: 200 μm. n = 5 per group

Cell surface markers were analyzed by flow cytometry as described previously [20]. The immunophenotype of OM-MSCs showed positive expression of CD73, CD90, and CD105, and negative expression of CCD31, CD34 and CD45. To assess the effects of OM-MSCs on pulmonary fibrosis, BLM-induced pulmonary fibrosis mice were generated and treated with OM-MSCs by tail vein injection on day 5, 10, and 15 after BLM administration. Ex vivo fluorescence imaging at 6 and 24 h post-injection showed a residency of OM-MSCs in the lung (Supplementary file 1: Fig. S1). Pulmonary CT showed reticular shadows, honeycomb changes and tractive bronchiectasis in both lung, and consolidation images were exhibited in BLM treatment group as revealed in transaxial and coronal sections. This fibrotic lesion was relieved by OM-MSC treatment (Fig. 1A-C). The BLM group also exhibited obvious thickening of the alveolar wall, and structural deformation of the lung parenchyma, and these changes were strikingly alleviated after OM-MSCs treatment, as revealed by hematoxylin and eosin (H&E) staining (Fig. 1D). Consistently, upon OM-MSCs treatment, BLM-induced mice showed decreased fibrosis by Ashcroft score (Fig. 1E). Meanwhile, Masson’s trichrome staining showed that the administration of OM-MSCs significantly reduced the accumulation of collagen (Fig. 1F-G). Furthermore, compared with the slow weight gain of PF mice treated with BLM, the weight change of the BLM + OM-MSC treatment group was approximately the same as the wild type control, and both increased steadily (Fig. S1). These phenomena indicate that OM-MSCs can effectively reduce the severity of BLM-induced PF in mice.

### Administration of OM-MSCs downregulates the expression of fibrotic and inflammatory factors in BLM-induced PF mouse model

**Fig. 2:**
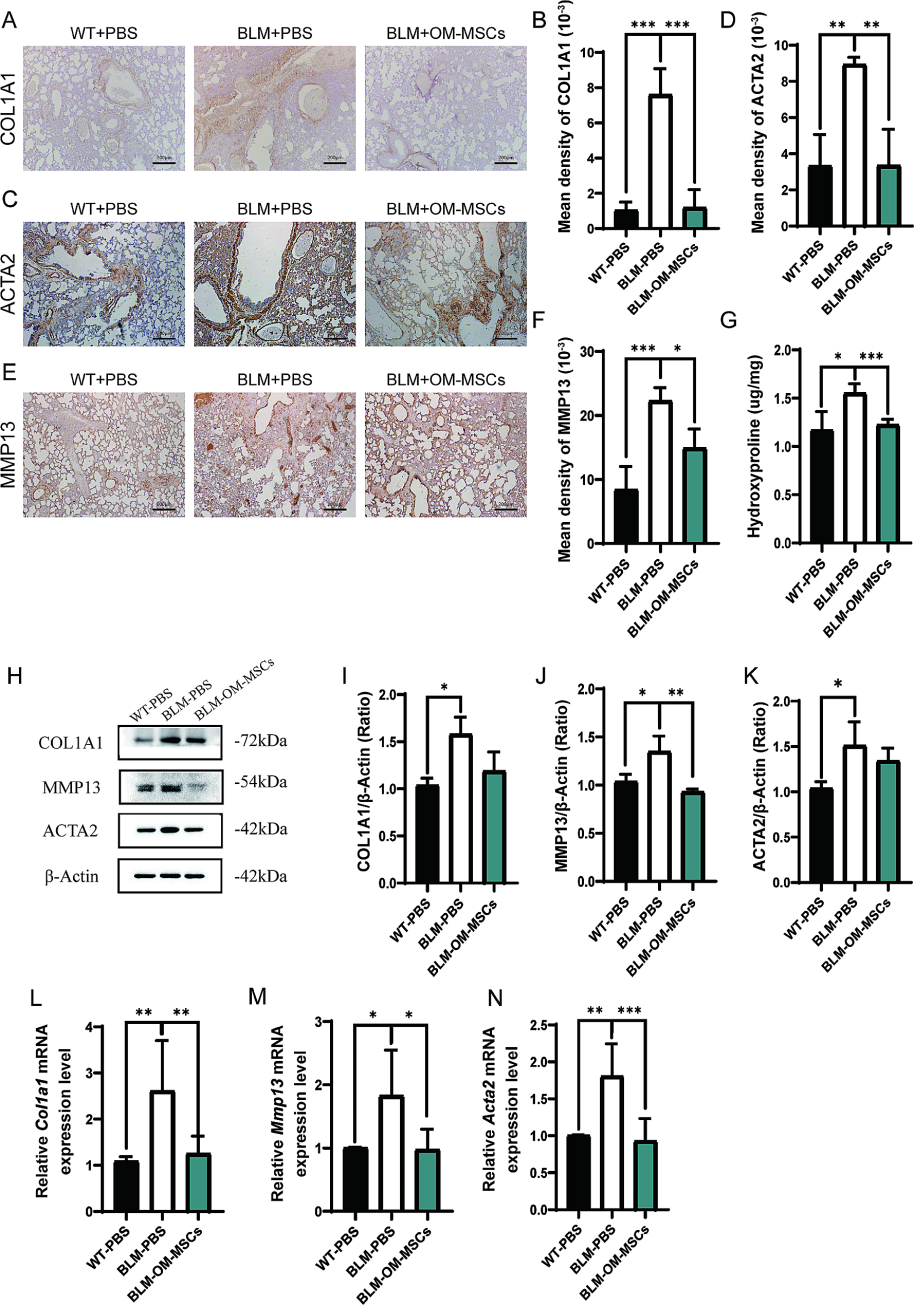
Administration of OM-MSCs downregulates the expression of fibrotic factors from BLM-induced pulmonary fibrosis. (**A-B**) Representative images and quantification of COL1A1 staining of lung tissues from control mice or BLM-induced mice with or without OM-MSCs treatment. Scale bar: 200 mm. n = 4 per group. (**C-D**) Representative images and quantification of ACTA2 staining of lung tissues from control mice or BLM-induced mice with or without OM-MSCs treatment. Scale bar: 200 mm. n = 4 per group. (**E-F**) Representative images and quantification of MMP13 staining of lung tissues from control mice or BLM-induced mice with or without OM-MSCs treatment. Scale bar: 200 mm. n = 4 per group. (**G**) Measurement of hydroxyproline depositions in lung tissue of control mice or BLM-induced mice with or without OM-MSC treatment. n = 4 per group. (**H-K**) Representative western blot analysis and quantification of COL1A1, MMP13, ACTA2. n = 3 per group. Full-length blots/gels are presented in Supplementary file 2. (**L-N**) QPCR analysis of *Col1a1*, *Mmp13*, *Acta2*. n = 6 per group

To determine the effects of OM-MSCs on fibrosis and inflammation in the BLM-induced lung fibrosis mouse model, the expression levels of fibrotic factors and inflammatory factors were determined. Immunohistochemical staining results of two fibrotic factors, collagen type I (COL1A1) and actin alpha 2, smooth muscle, aorta (ACTA2/α-SMA), were obviously increased after BLM treatment. These factors were markedly decreased after OM-MSC transplant, indicating that collagen deposition in the lungs of the mice was obviously relieved by OM-MSC transplantation (Fig. 2A-D). Furthermore, the expression level of matrix metallopeptidase 13 (MMP13) which mediates matrix remodeling in vivo, was significantly increased after BLM treatment, and downregulated after OM-MSCs administration (Fig. 2E-F), consistent with data that MMP13 was upregulated in PF patients. Compensatory MMP13 expression is to antagonize the collagen deposition and contributes to the development of honeycomb cysts [23, 24]. In addition, we evaluated the collagen accumulation by measuring hydroxyproline, which is the main component of collagen. Hydroxyproline significantly reduced after OM-MSC treatment in BLM-induced PF mouse model (Fig. 2G). The protein levels of COL1A1, ACTA2, MMP13, as well as mRNA levels of *Col1a1, Acta2, Mmp13*, were lower in BLM + OM-MSC treatment group as compared with BLM treatment group (Fig. 2H-N).

**Fig. 3:**
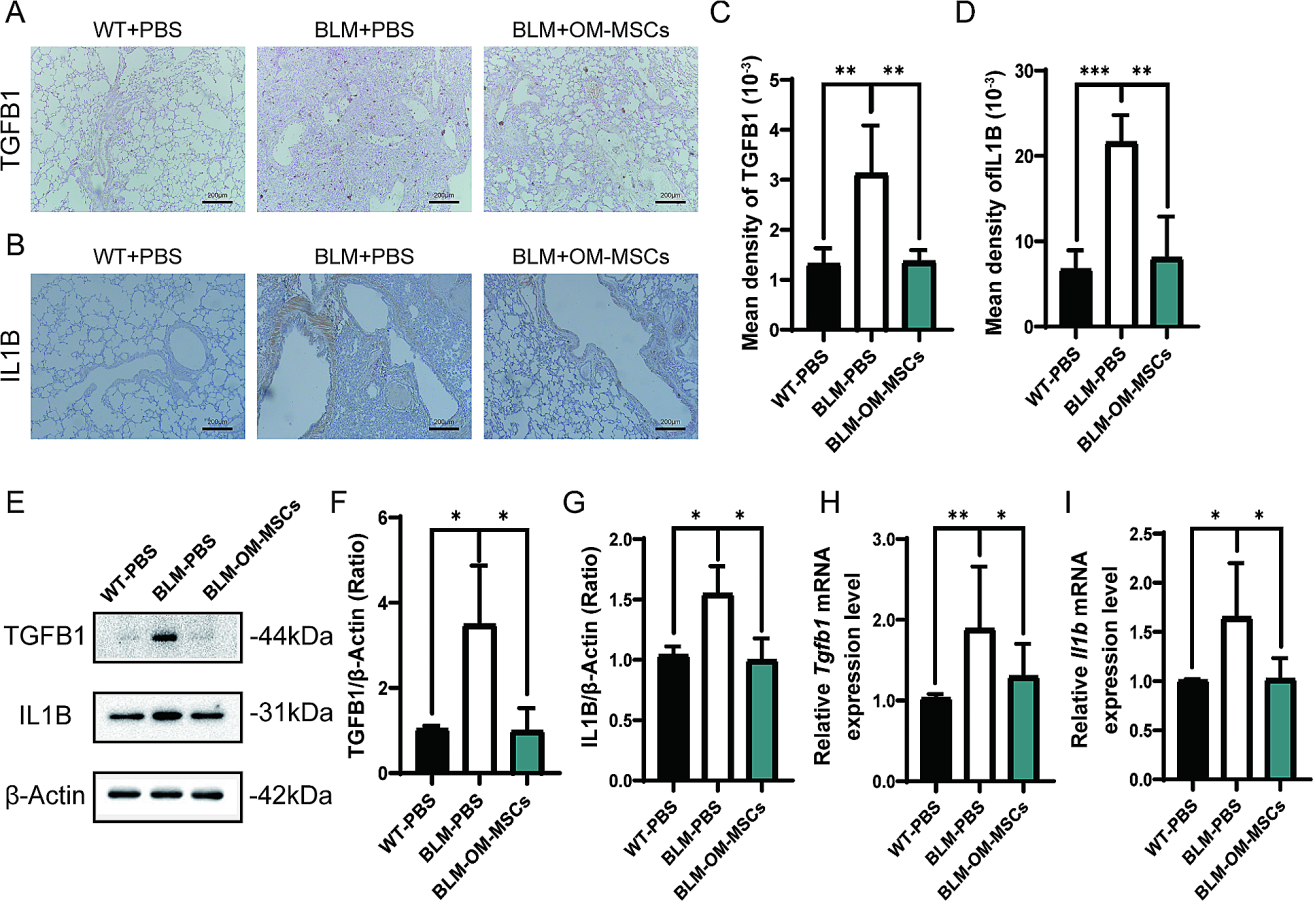
Administration of OM-MSCs downregulates the expression of inflammatory factors from BLM-induced pulmonary fibrosis. (**A-D**) Representative images and quantification TGFB1, IL1B staining of lung tissues from control mice or BLM-induced mice with or without OM-MSCs treatment. Scale bar: 200 mm. n = 4 per group. (**E-G**) Representative western blot analysis and quantification of TGFB1, IL1B. n = 3 per group. Full-length blots/gels are presented in Supplementary file 2. (**H-I**) QPCR analysis of *Tgfb1*, *Il1b*. n = 6 per group

Moreover, we determined the expression levels of inflammatory factors. As reveled by immunohistochemistry images, western blot, and qRT-PCR, the levels of TGFB1 and IL1B in the lungs were significantly upregulated after BLM administration as compared with the control group, but the expression levels of these inflammatory factors in the OM-MSC treatment group were restored to levels similar to their normal conditions (Fig. 3A-I). In summary, the transplant of OM-MSCs can effectively inhibit up-regulated inflammatory factors and the accumulation of collagen.

### Administration of OM-MSCs modulates monocyte-derived macrophage populations in the lung

**Fig. 4:**
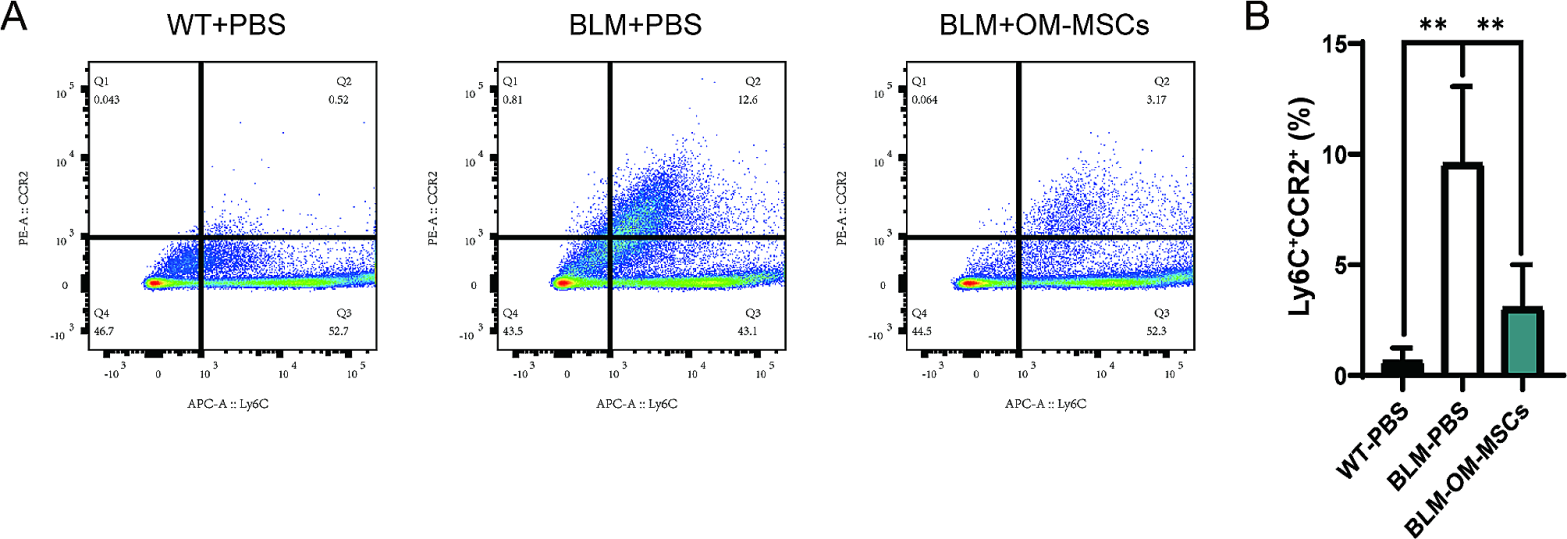
Administration of OM-MSCs promotes pro-resolution LY6C1. ^low^ monocyte generation on bleomycin-induced pulmonary fibrosis. (**A-B**) Flow cytometry analysis of LY6C1 monocytes from lung tissues of control mice or BLM-induced mice with or without OM-MSCs treatment

To investigate the ways in which OM-MSCs affect the above cytokine changes during pulmonary fibrosis, we analyzed the changes in the immune cell population in the lung, mainly infiltrating monocytes. Flow cytometric analysis for proinflammatory classical monocytes (LY6C1^hi^ CCR2^+^cells) and nonclassical monocytes (LY6C1^low^ CCR2^−^cells) revealed that the ratio of proinflammatory monocytes to nonclassical monocytes in the lung increased after BLM-induced lung injury compared with control mice, while the opposite results appeared following OM-MSCs transplant, suggesting OM-MSCs were able to alter the proportion of monocytes infiltrated in the lung to alleviate inflammation and fibrosis (Fig. 4).

**Fig. 5:**
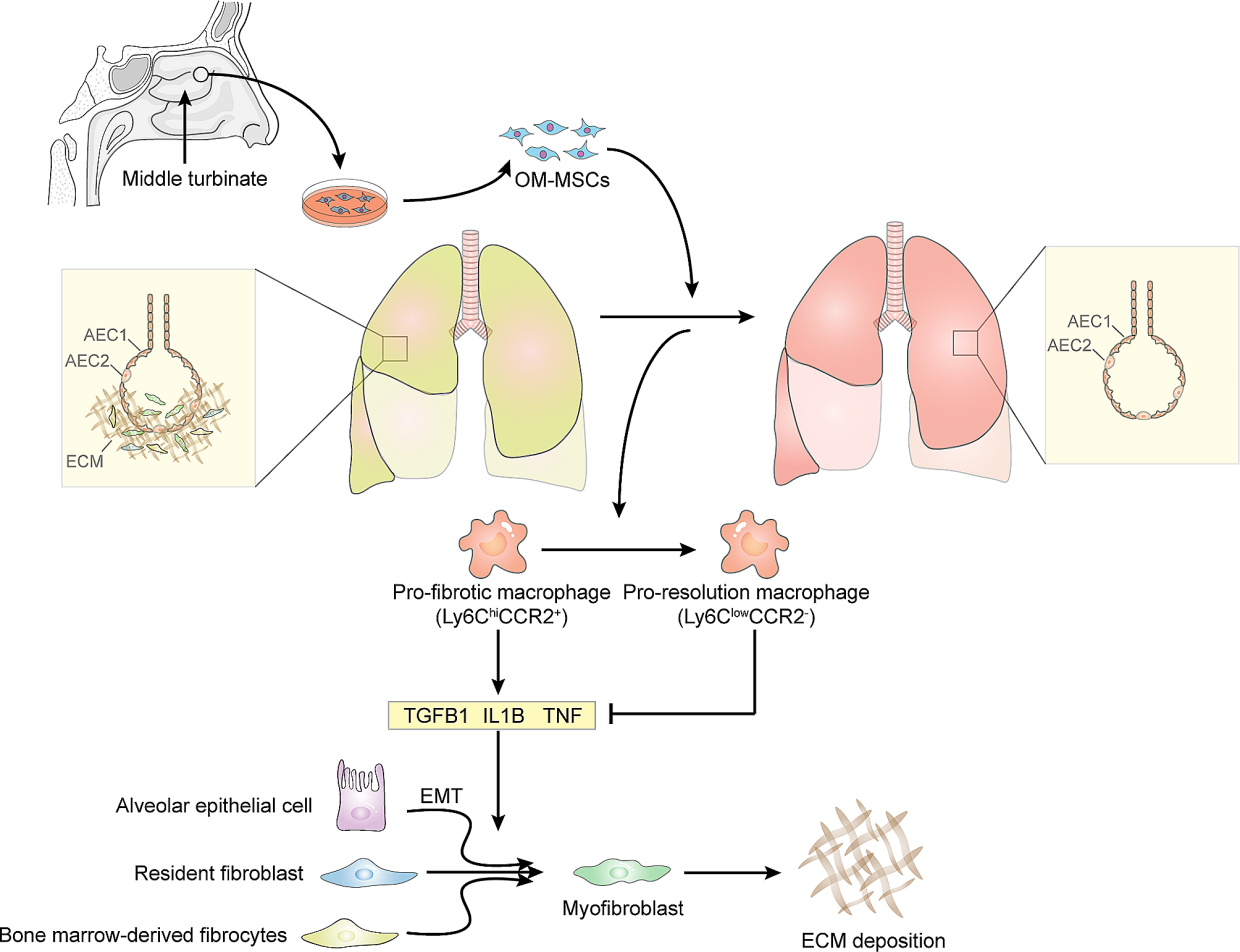
OM-MSCs ameliorate pulmonary fibrosis by modulating monocyte-derived macrophage phenotype. Pro-fibrotic macrophage can secrete TGF-β, TNF, IL1B and other factors to increase the number of myofibroblasts through EMT, thus producing more ECM and leading to fibrosis. OM-MSCs can reverse this process by altering the monocyte-derived macrophage phenotype

In conclusion, transplant of OM-MSCs may alleviate the PF by increasing the pro-resolution macrophage, inhibiting the inflammatory factors secretion, decreasing the fibrotic factors (Fig. 5).

## Discussion

Although most patients with PF exhibit a slow, progressive course over several years, due to delayed diagnosis and acute exacerbations caused by complications, several retrospective longitudinal studies indicate that the average life expectancy of these patients after diagnosis is reported to be 3–5 years [25–28]. It has been long appreciated that PF can be relieved by MSCs transplant. However, the source of MSCs, and the underlying mechanism remains challenging. Our study provides the first evidence that transplant of OM-MSCs can attenuate pulmonary fibrosis and induce a pro-resolution phenotype by increasing LY6C1^low^ macrophages, inhibiting inflammation, and enhancing degradation of ECM.

Although previous studies have reported the benefits of MSCs transplant on PF [29]. But the source of MSCs limits the application. Human umbilical cord blood MSCs (UCB-MSCs) are not available for adults, BM-MSCs have been found to be more antigenic, have lower proliferative capacity, and have weaker paracrine potency when compared to UBC-MSCs [30], MSCs factory production lost many characteristics of the stem cells [31]. Since the limitation of adult MSCs, allotransplant is carried out instead of autologous transplant, however, the use of anti-rejection regimes has severe side effects for PF patients. It is promising for PF treatment while we get over the obstacle of procurement of enough autologous MSCs. Herein, we reported that OM-MSCs are an optional source for PF, which was first defined by Huard et al. (1998) and characterized as CD73 + CD90 + CD105 + CD34- CD45- CD31- [32, 33]. It has the advantages of easy accession and high versatility. Most importantly, OM-MSCs could be used for autologous transplant and had beneficial effects for the treatment of Parkinson’s disease, rheumatoid arthritis and other autoimmune diseases [33–35]. Therefore, OM-MSCs can maintain the features of MSCs, proliferate in vitro, providing enough number of OM-MSCs for treatment of PF with simple surgical procedures and in vitro expansion, without any harmful side effects on the patients.

MSCs home to sites of injury, inhibit inflammation, and contribute to epithelial tissue repair. Their use has been suggested as a therapy for the treatment of PF [29]. However, the underlying mechanism of MSC treatment of PF remains unknown. The mechanistic studies of PF show that MSCs may inhibit the secretion of cytokines [36]. Consistent with previous studies, our present work demonstrated that OM-MSCs inhibit the secretion of pro-inflammatory cytokines, enhance degradation of ECM, increase LY6C1^low^ macrophages, which is a restorative macrophage subpopulation switched from pro-inflammatory LY6C1^hi^ subset and crucial for remodeling of fibrosis [13].

The limitation of our study is that the comparison of effects between OM-MSCs and other PF regimes was not evaluated. MSCs derived from umbilical cord, bone marrow, and adipose tissue have all been used in studies investigating their impact on pulmonary fibrosis [37, 38]. However, our research demonstrates that OM-MSCs exhibit significant therapeutic efficacy in a bleomycin-induced pulmonary fibrosis model, and OM-MSCs are more easily obtained and suitable for autologous transplantation. The comparison of systemically (intravenous (IV) or intraperitoneal (IP)) vs. intratracheal (IT) MSCs administration was also another issue that we did not measure. A comparison study found that intravenous administration is more rapid and more effective in exerting its effects compared to intratracheal administration [39]. In a pre-clinical study, results showed that IT administration was more efficacious at reversing lung fibrosis at a four-fold lower dose of MSCs [40]. In our study, we have determined that allotransplant of OM-MSCs (IV) is easily operated, appeared to be effective and safe in the short-term; however, ongoing follow-up of these subjects would be necessary before conclusions regarding long-term safety could be made.

The range of pulmonary fibrotic diseases observed in coronavirus infection, encompassing fibrosis linked to organizing pneumonia to severe acute lung injury, leading to the development of extensive fibrotic changes [41]. Despite many patients surviving the acute phase of the disease and possibly being discharged, a significant proportion eventually succumb to progressive pulmonary fibrosis [42]. The severe respiratory consequences of the coronavirus disease 2019 (COVID-19) pandemic have prompted urgent need for novel therapies for lung fibrosis to resolve the social problem as important as blocking coronavirus transmission. MSCs administration can significantly reduce respiratory virus [43]. Autologous transplant of OM-MSCs may benefit these patients with pulmonary fibrosis. It is a promising and newly attractive source of MSCs for fibrosis treatment.

## Conclusions

Our study presented here demonstrate that transplant of OM-MSCs can attenuate pulmonary fibrosis by inhibiting inflammation and enhancing degradation of ECM, which may be achieved by increasing LY6C1^low^ macrophages in the lung. This result may provide a new avenue for the treatment of pulmonary fibrosis in the context of coronavirus disease.

### Electronic supplementary material

Below is the link to the electronic supplementary material.

Supplementary Material 1

Supplementary Material 2

## Data Availability

The datasets obtained and analyzed for this study will be available from the corresponding author at a reasonable request.

## Abbreviations

ACTA2/a-SMA: actin, alpha 2, smooth muscle, aorta
BLM: bleomycin
COL1A1: collagen, type I, alpha 1
ECM: extensive extracellular matrix
IL1B: interleukin 1 beta
LY6C1: lymphocyte antigen 6 complex, locus C1
MMP13: matrix metallopeptidase 13
MSCs: mesenchymal stem cells
PF: pulmonary fibrosis
TGFB1: transforming growth factor, beta 1
