# Supplementary material for: Olfactory mucosa mesenchymal stem cells alleviate pulmonary fibrosis via the immunomodulation and reduction of inflammation
Source: BMC Pulm Med. 2024 Jan 5;24:14. doi: 10.1186/s12890-023-02834-5 (PMC10768423; doi:10.1186/s12890-023-02834-5)
Supplement: Supplementary file 1 — Supplementary Material 1 [file 12890_2023_2834_MOESM1_ESM.pdf]

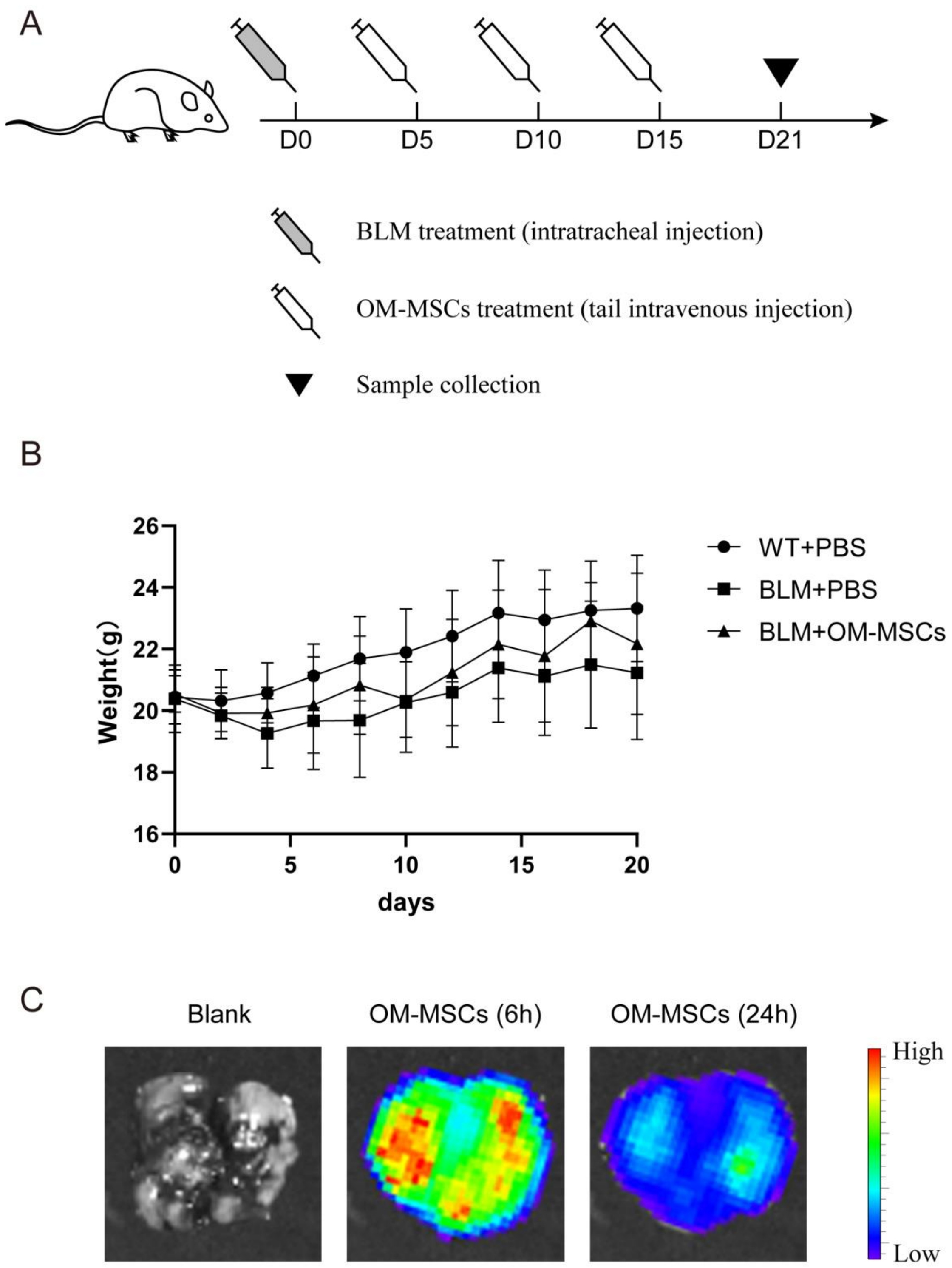

Supplementary File. 1 | (A) Schematic of generating BLM-induced PF. **(B)** Body weight of control mice or BLM-induced mice with or without OM-MSCs treatment. n = 10 per group. **(C)** Fluorescence image of distribution of OM-MSCs.
