# Supplementary figures and images for: Olfactory mucosa mesenchymal stem cells alleviate pulmonary fibrosis via the immunomodulation and reduction of inflammation
Source: BMC Pulm Med. 2024 Jan 5;24:14. doi: 10.1186/s12890-023-02834-5 (PMC10768423; doi:10.1186/s12890-023-02834-5)

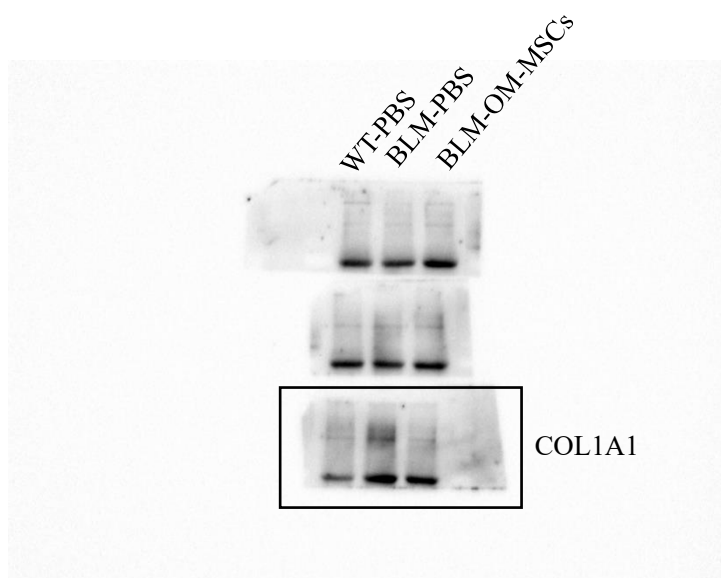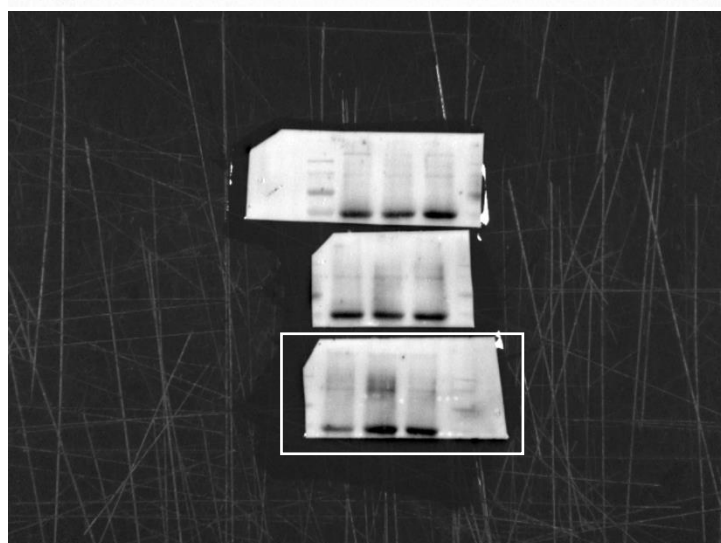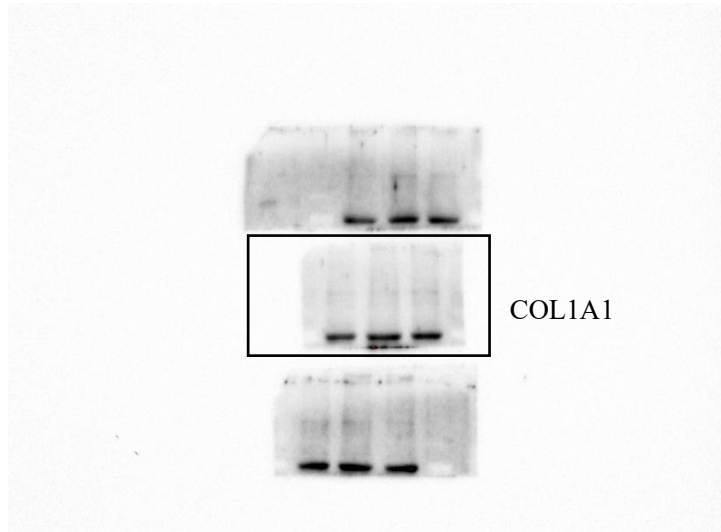

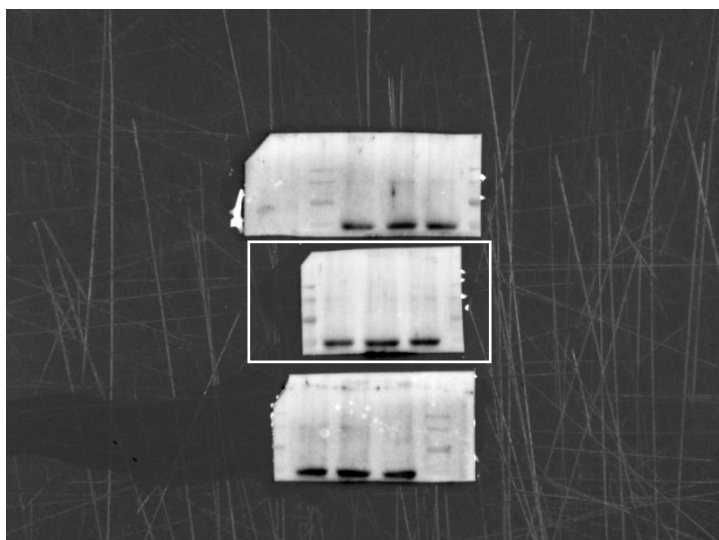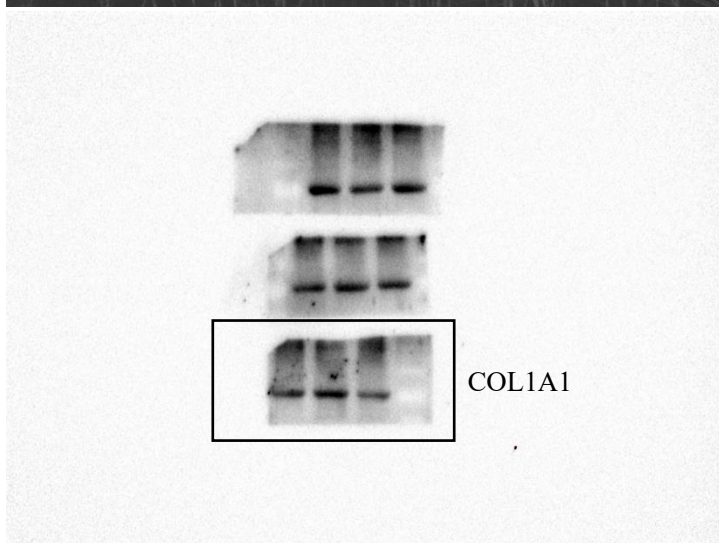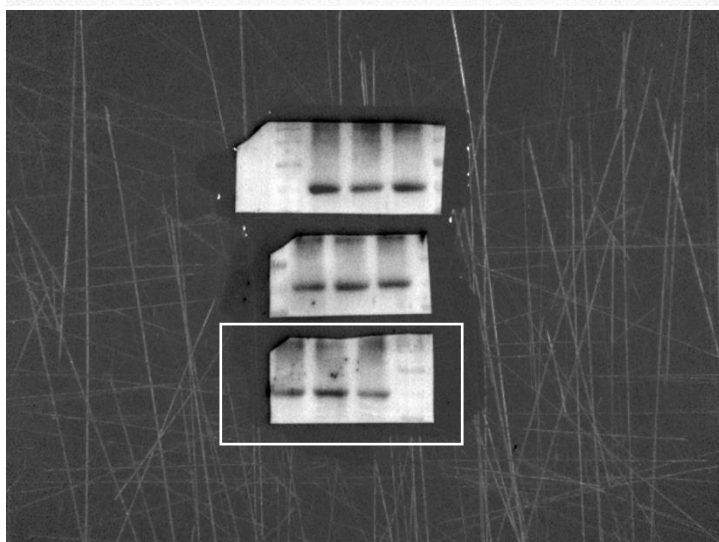

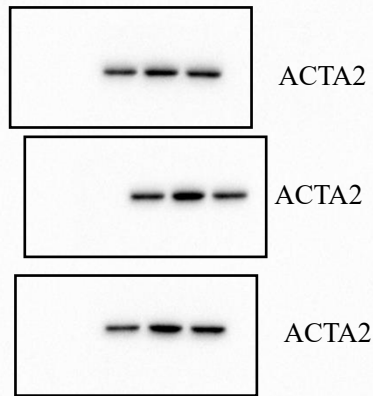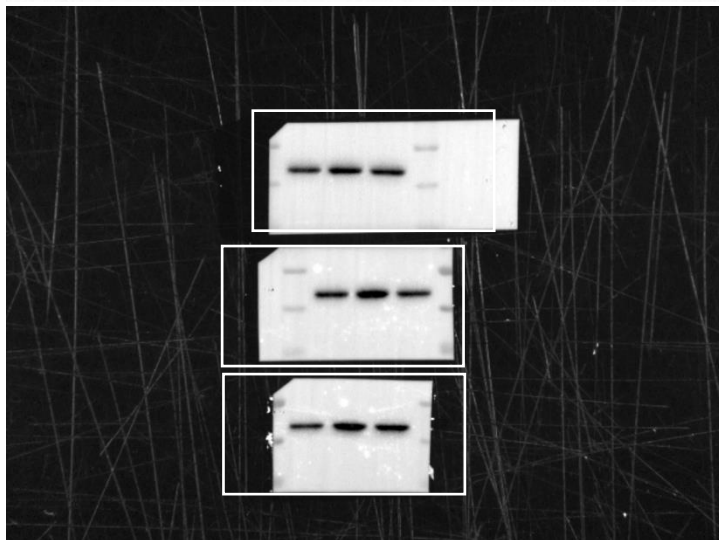

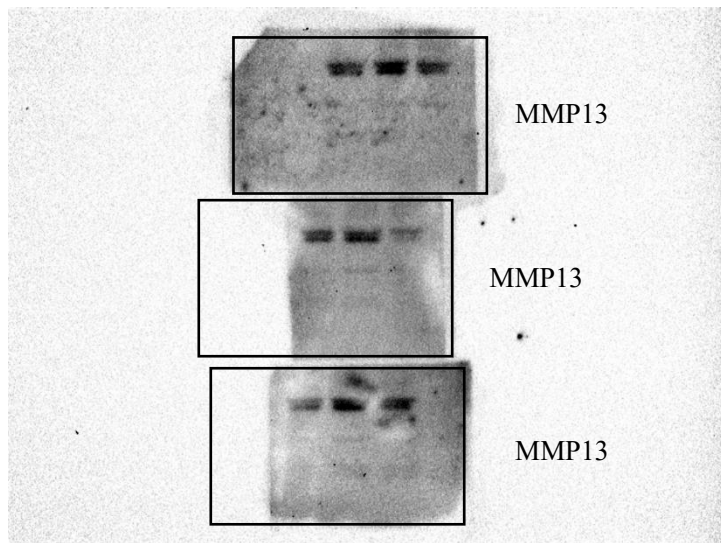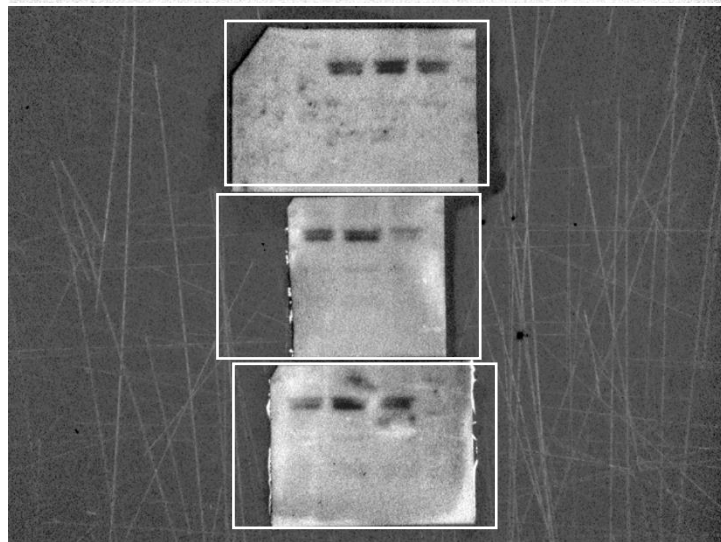

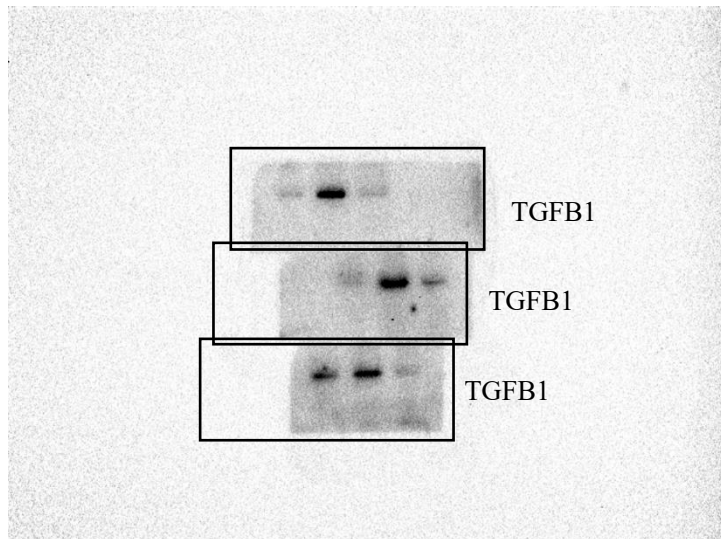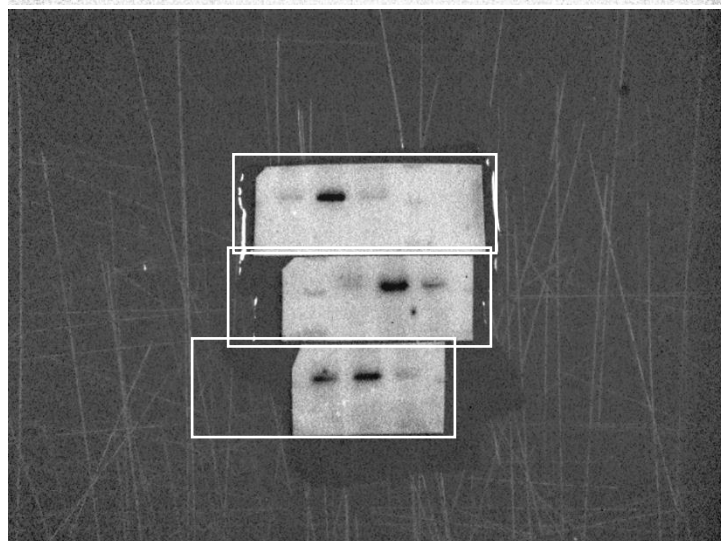

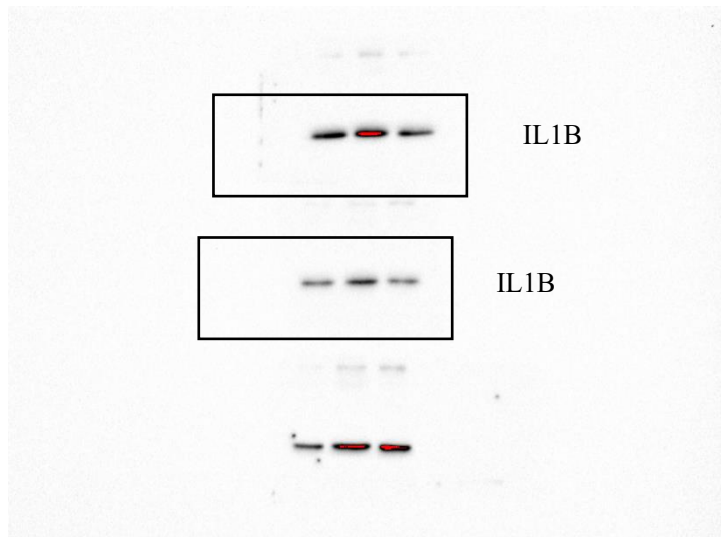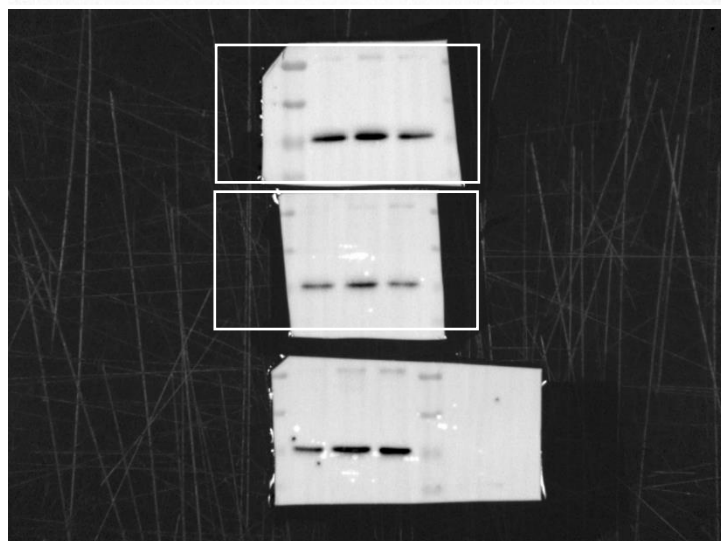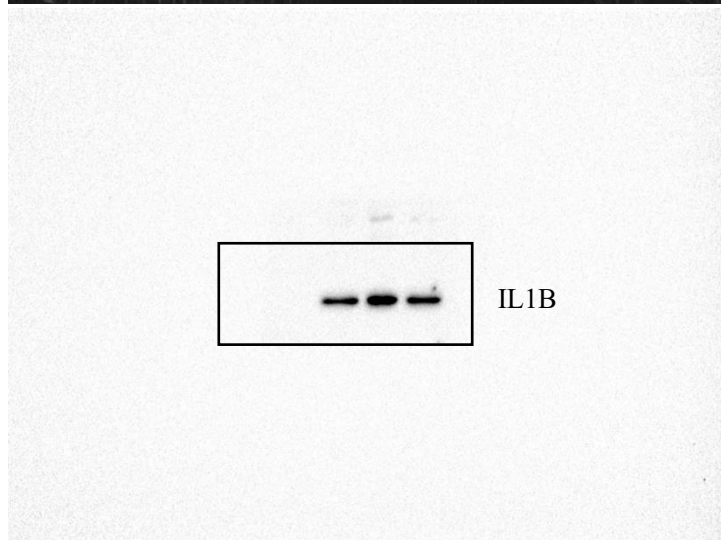

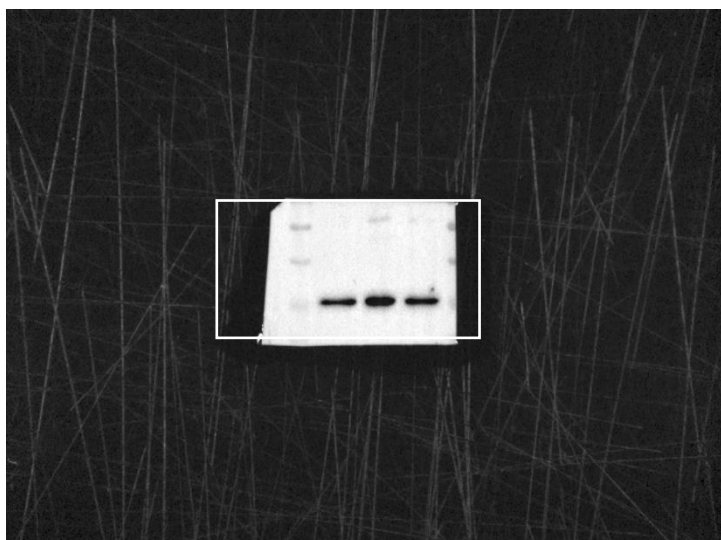

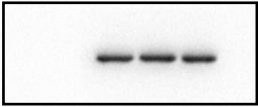

$\beta$ -Actin

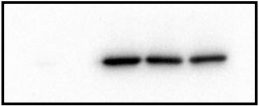

$\beta$ -Actin

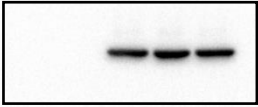

$\beta$ -Actin

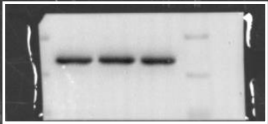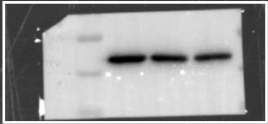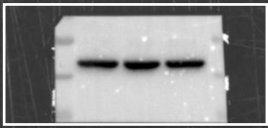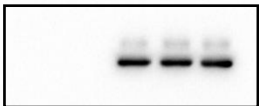

$\beta$ -Actin

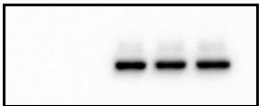

$\beta$ -Actin

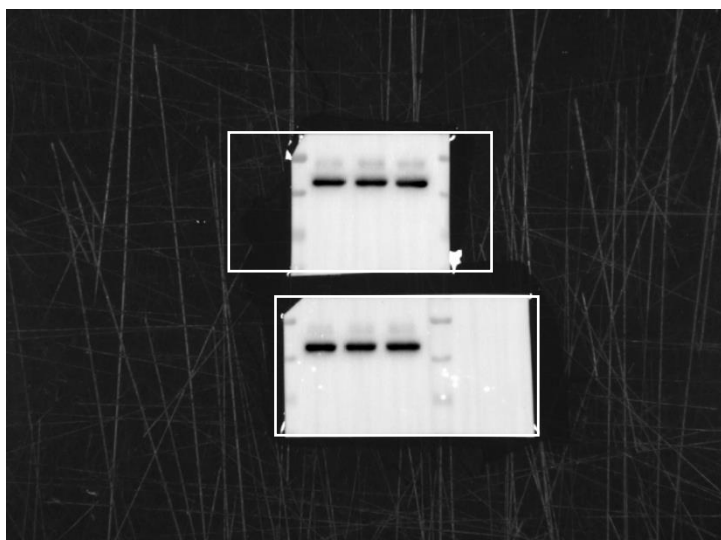

Supplement: Supplementary file 2 — Supplementary Material 2 [file 12890_2023_2834_MOESM2_ESM.pdf]
